# Aggravated brain injury after neonatal hypoxic ischemia in microglia-depleted mice

**DOI:** 10.1186/s12974-020-01792-7

**Published:** 2020-04-11

**Authors:** Shunichiro Tsuji, Elena Di Martino, Takeo Mukai, Shoko Tsuji, Takashi Murakami, Robert A. Harris, Klas Blomgren, Ulrika Åden

**Affiliations:** 1 Department of Women’s and Children’s Health, Karolinska Institutet, Stockholm, Sweden; 2 Department of Obstetrics and Gynecology, Shiga University of Medical Science, Seta Tsukinowa-cho, Otsu City, Shiga 520-2192 Japan; 3 Applied Immunology and Immunotherapy, Department of Clinical Neuroscience, Karolinska Institutet, Center for Molecular Medicine, Karolinska Hospital Solna, Stockholm, Sweden; 4 Department of Pediatric Oncology, Karolinska University Hospital, Stockholm, Sweden; 5 Department of Neonatal Medicine, Karolinska University Hospital, Stockholm, Sweden

**Keywords:** Microglia, Hypoxic-ischemic encephalopathy, Neonate, Ischemic stroke

## Abstract

**Background:**

Neuroinflammation plays an important role in neonatal hypoxic-ischemic encephalopathy (HIE). Although microglia are largely responsible for injury-induced inflammatory response, they play beneficial roles in both normal and disease states. However, the effects of microglial depletion on neonatal HIE remain unclear.

**Methods:**

Tamoxifen was administered to Cx3cr1^CreER/+^Rosa26^DTA/+^ (microglia-depleted model) and Cx3cr1^CreER/+^Rosa26^DTA/−^ (control) mice at P8 and P9 to assess the effect of microglial depletion. The density of microglia was quantified using Iba-1 staining. Moreover, the proportion of resident microglia after the HI insult was analyzed using flow cytometric analysis. At P10, the HI insult was conducted using the Rice-Vannucci procedure at P10. The infarct size and apoptotic cells were analyzed at P13. Cytokine analyses were performed using quantitative polymerase chain reaction and enzyme-linked immunosorbent assay (ELISA) at P13.

**Results:**

At P10, tamoxifen administration induced > 99% microglial depletion in DTA^+^ mice. Following HI insult, there was persisted microglial depletion over 97% at P13. Compared to male DTA^−^ mice, male DTA^+^ mice exhibited significantly larger infarct volumes; however, there were no significant differences among females. Moreover, compared to male DTA^−^ mice, male DTA^+^ mice had a significantly higher density of TUNEL^+^ cells in the caudoputamen, cerebral cortex, and thalamus. Moreover, compared to female DTA^−^ mice, female DTA^+^ mice showed a significantly greater number of TUNEL^+^ cells in the hippocampus and thalamus. Compared to DTA^−^ mice, ELISA revealed significantly lower IL-10 and TGF-β levels in both male and female DTA^+^ mice under both normal conditions and after HI (more pronounced).

**Conclusion:**

We established a microglial depletion model that aggravated neuronal damage and apoptosis after the HI insult, which was predominantly observed in males.

## Background

Neonatal hypoxic-ischemic encephalopathy (HIE) is a major worldwide cause of neonatal death and long-term disability, including mental retardation, visual motor or visual perceptive dysfunction, hyperactivity, cerebral palsy, and epilepsy. HI insults are known to induce inflammatory reactions within hours in rodent models with microglia playing an important role [1–5].

Microglia are resident cells in the brain that are the first responders to ischemia. These cells engage in intimate cross-talk with other intrinsic brain cells and infiltrating leukocytes from the periphery that enter the brain through the compromised blood-brain barrier (BBB) [6–8]. In response to brain ischemia, microglia initiate reactive oxygen species generation, antigen presentation, phagocytosis, and the production of inflammatory mediators, including interleukin (IL)-1β, tumor necrosis factor (TNF)-α, IL-6, and matrix metalloproteinases [8–10]. Contrastingly, microglia plays an anti-inflammatory role by producing factors such as IL-4, IL-10, and transforming growth factor-β (TGF-β) during the brain inflammation-resolution and on return to homeostatic surveillance [10, 11]. There have been extensive studies on the active role of microglia in ischemic brain injury in the adult brain [12–17]. However, the response to HIE largely differs between the adult and neonatal brain [7, 18–21].

The specific role of microglia can be investigated using microglial depletion models [22–32]. Most studies on microglial depletion employ pharmacological strategies involving the colony-stimulating factor 1 receptor (CSF1R), including PLX33397 or PLX5622 [33]. However, this procedure requires at least 2 weeks for microglia depletion. Therefore, there is a need for alternative methods for assessing the role of microglia depletion in neonatal HIE.

Neonatal HIE is known to show sexual dimorphism. A previous study on 1,864,766 infants born in California reported that males have a 1.27-fold higher risk (95% CI, 1.2–1.4) of HIE [34]. A previous study reported that rodent models had no between-sex differences in the infarct size on the first day after HI; however, male neonates presented significantly larger infarct sizes 3 days after HI [35]. Moreover, with respect to long-term developmental outcomes, males are more vulnerable than females [36, 37]. Similarly, there are sex differences in the microglial number, morphology, migration, and phagocytic activity [38–40].

We aimed to establish a model for studying the effects of microglial depletion on experimental neonatal HIE, including the infarct size, apoptotic cells, and cytokine response. Moreover, we aimed to determine sex differences in the effects of post-HI microglial depletion.

## Materials and methods

### Animals

**Fig. 1:**
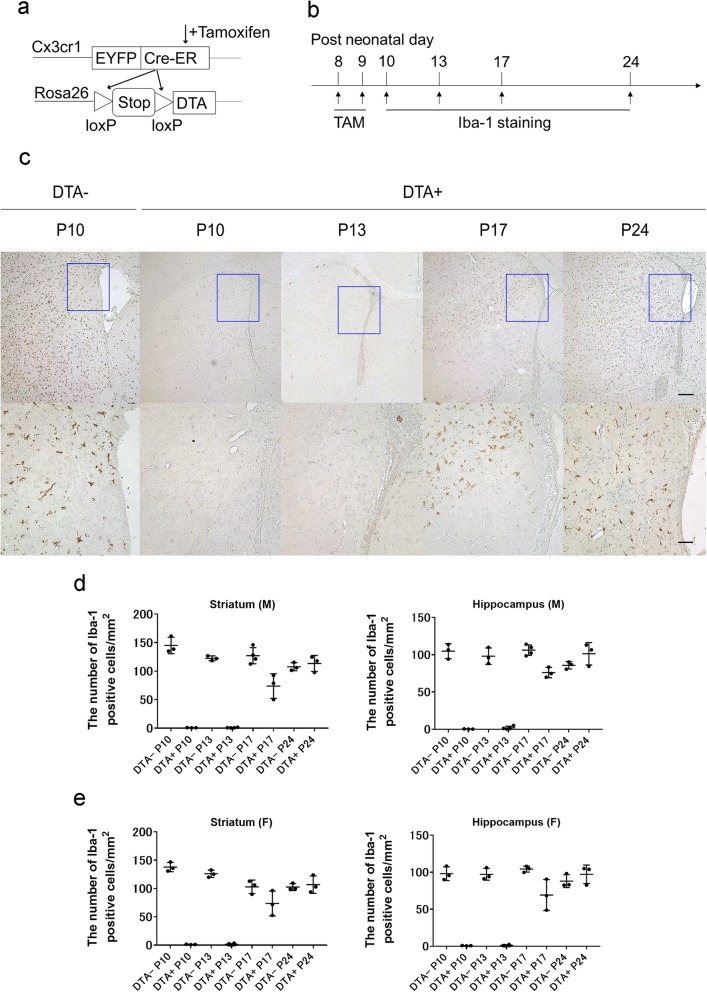
Microglial depletion and repopulation following tamoxifen (TAM) administration. **a** Scheme of the genetic design. **b** Scheme of the repopulation study. **c** Representative Iba-1 staining slides for microglia in the striatum level at each time point. Scale bar indicates 200 μm in the upper column and 50 μm in the lower column. The square in the upper column indicates higher magnification in the lower column. **d** The Iba-1^+^ cell density in the striatum level and hippocampus level in male mice. **e** The Iba-1^+^ cell density in the hippocampus level in female mice

This study was approved by the ethics committee at Karolinska Institutet, Stockholms Norra djurförsöksetiska nämnd (approval number: N94/15 and N126/16) and was conducted according to the relevant guidelines and regulations (Swedish Animal Welfare Act 1988:543), as well as the ARRIVE guidelines for animal experiments. We used *Cx3cr1*^*EYFP-CreER*^ mice, which express a Cre-ERT2 fusion protein and enhanced yellow fluorescent protein (EYFP), and Rosa26^DTA+/-^ mice, which carry a *loxP*-flanked stop cassette associated with an attenuated diphtheria toxin [41]. Crossbreeding of these two strains produced *Cx3cr1*^*CreER-EYFP+*^*/Rosa26*^*DTA+/*−^ mice (DTA+ mice, microglia-depleted mice) and *Cx3cr1*^*CreER-EYFP+*^*/Rosa26*^*DTA−/−*^ mice (DTA^*−*^ mice, control mice) (Fig. 1a). All the mice were housed in a humidity-controlled room with a 12-h light-dark cycle and ad libitum access to food and water.

### Tamoxifen administration

Tamoxifen was purchased from Sigma-Aldrich (Cat. #T5648), dissolved in corn oil (8 mg tamoxifen/1 ml corn oil) at 70 °C, and intraperitoneally (50 mg/kg) injected to the mice at P8 and P9.

### Induction of hypoxic-ischemic encephalopathy

HIE induction was performed without knowing the genotype. Unilateral HI was induced at P10 using the modified Rice-Vannucci model [42–44]. After sedation with isoflurane (4% for induction and 2% for maintenance) and local anesthesia with Marcaine (Astra Zeneca), the left common carotid artery was isolated and occluded through 8-Watt electronic coagulation (Figure S1). The skin incision was sutured with 6-0 silk surgical thread and infiltrated with additional local anesthesia. All mice were subjected to ischemic surgery within < 5 min. Pups were returned to the dam for 1 h with subsequent placement in a hypoxic chamber (BioSpherix, NY, USA) with 10% O_2_ in 90% N_2_ for 1 h. For mice in the sham group, the carotid artery was isolated but not cauterized. For injury evaluation and cytokine analysis, 40 and 64 pups underwent the HI insult, respectively. The sham group was included 41 pups.

### Immunohistochemistry

The mice were anesthetized using an injection of 50 mg/kg pentobarbital (APL, Sweden) and transcardially perfused with phosphate-buffered saline (PBS) followed by 4% paraformaldehyde (PFA) at P10, P13, P17, and P24 for a repopulation study and at P13 after HI for evaluation of the infarction size. The removed brains were fixed in 4% PFA for 24 h, dehydrated, embedded in a paraffin block, and coronally sectioned to 5-μm-thick slices using a sliding microtome. Six levels of each brain were collected as previously reported [18, 45, 46]. The first level was obtained corresponding to the bregma 1.3 mm in the adult mouse brain with every 100th section being collected and deparaffinized. After deparaffinization, antigen retrieval was performed in 10 mM citric buffer (pH 6.0) for 10 min. After blocking, the sections were exposed to primary antibodies in a humidity chamber box overnight at 4 °C. The primary antibodies were those against rabbit Iba-1 obtained from Wako (#1919741) and diluted at 1:1000, mouse microtubule-associated protein-2 (MAP-2) obtained from Sigma (#M4403) and diluted at 1:1000, rabbit anti glial fibrillary acidic protein (GFAP) diluted at 1:500 (#ab5804), chicken anti-green fluorescent protein obtained from Abcam (#ab13970) and diluted at 1:500, and mouse anti myelin basic protein (MBP) obtained from Covance and diluted 1:1000. After washing, biotin-conjugated donkey anti-rabbit secondary antibody (Jackson ImmunoResearch) diluted at 1:1000 was applied for Iba-1 while the VECTOR M.O.M.™ Immunodetection Kit was used for MAP-2. After blocking endogenous peroxidase using 0.3% H_2_O_2_, the sections were visualized using the VECTASTAIN Elite ABC-Peroxidase Kit and 3,3′-diaminobenzidine. For GFAP, biotinylated donkey anti-rabbit secondary antibody was applied for 1 h prior to incubation with VECTASTAIN® Elite ABC-Peroxidase Kit. For immunofluorescence analyses, the following secondary antibodies were used: donkey anti-rabbit IgG Alexa Fluor 555 (1:1000) and donkey anti-chicken Alexa Fluor 488 (1:1000). Nucleus staining was performed using Hoechst 33342 (1:1000, ThermoFisher). Coverslips were mounted onto slides using antifade reagent ProLong Gold (Molecular probes/Life technologies).

### Iba-1^+^ cell counting

Sections were examined using Zeiss Axio Imager M2 (Carl Zeiss, Göttingen, Germany). The microglia number was determined by counting the Iba-1-positive cells in the whole hemisphere. The total Iba-1^+^ cell number was calculated using the fractionator option in Stereo Investigator software (MicroBrightField, Colchester, Vt., USA). Briefly, each section was divided into 200 approximately equal squares (400 × 400 μm, sampling grid). Subsequently, the number of Iba-1^+^ cells with a complete cell body was counted in each 100 × 100 μm red-green square (counting frame) using a × 20 air objective. The software allowed estimation of the total cell number and the whole coronal section area. Slides from the DTA^+^ mice at P10, P13, and P17 showed few unevenly distributed microglial cells; therefore, all the Iba-1^+^ cells were directly counted without using a fractionator. Based on these data, the Iba-1^+^ cell density was calculated.

### Fluorescence-activated cell sorting

Mice were sacrificed using an intraperitoneal injection of 50 mg/kg pentobarbital (APL, Sweden) and transcardially perfused with PBS. After 5 min of perfusion, the brains were extracted and the olfactory bulbs and cerebellum removed. Subsequently, the right and left hemispheres were separated and individually processed. Next, the tissue was minced with a scalpel and digested for 30 min at 37 °C with Hank’s Balanced Salt Solution (HBSS) containing 0.02% DNase 1 (Sigma-Aldrich) and 0.005% collagenase (Sigma-Aldrich). The reaction was stopped using cold HBSS containing 0.01% ethylenediaminetetraacetic acid (EDTA); subsequently, the samples were strained and centrifuged at 4 °C (300*g*, 10 min). The pellets were resuspended in 38% percoll solution and centrifuged at 4 °C (800*g*, 10 min). After careful removal of the supernatant, the pellets were rinsed with PBS and transferred to a V-bottom plate for staining. The samples were incubated for 20 min at 4 °C with PBS containing CD11b (1:100, M1-70, BioLegend), CD45 (1:1000, 30-F11, BioLegend), and LIVE/DEAD™ Fixable Near-IR Dead Cell Stain Kit (Invitrogen) and rinsed before analysis. The cell numbers were determined using the Gallios flow cytometer (Beckman Coulter) and analyzed using Kaluza software (Beckman Coulter).

### Injury evaluation

At P13, tissue loss was assessed by determining the MAP-2-stained area as previously described [18, 45]. First, the MAP-2-positive area was separately measured in each hemisphere using Stereo Investigator software (MicroBrightField, USA). Second, the difference in the areas was calculated by subtracting the staining area in the contralateral hemisphere from that in the ipsilateral hemisphere. Next, the total tissue loss volume was calculated based on the Cavalieri principle using the following formula: *V* = ΣA·*P*·*T* (where *V* is the total volume, ΣA is the sum of the areas measured, *P* is the inverse of the sampling fraction, and *T* is the section thickness). This evaluation was performed with genotype blinding.

### TUNEL and NeuN double staining

After deparaffinization, antigen retrieval was performed in 10 mM citric buffer (pH 6.0) for 15 min. The sections were incubated with 5% normal donkey serum and 0.3% Triton X-100 in PBS for 30 min at room temperature followed by rabbit monoclonal anti-NeuN antibody (1:100, MABN140, Millipore Corpo, CA, USA) at 4 °C overnight. After washing out the primary antibody, donkey anti-rabbit Alexa Fluor 488 (1:300, A-212206, Thermo Fisher Scientific) was applied for 2 h at room temperature. The sections were rinsed with PBS followed by the terminal deoxynucleotidyl transferase-dUTP nick end labeling (TUNEL) (Click-iT Plus TUNEL Assay Kit, C10618, Thermo Fisher Scientific Europe BV) assay according to the manufacturer’s protocol. Stereological analysis was performed using Stereo Investigator software (MicroBrightField, USA). The number of TUNEL^+^ cells was counted under × 20 magnification [47, 48]. First, each region’s contour (striatum, hippocampus, cortex, and thalamus) was traced using a × 5 air objective. The counting frame and grid size were both set to 150 × 150 μm, and the number of TUNEL-positive cells was counted using a × 20 air objective. Cell counting was performed by an investigator blinded to the genotype. Fluorescent images were obtained using a LSM700 laser scanning confocal microscope (Axio-observer Z1; Carl Zeiss microscopy, Germany) and analyzed using the software ZEN 2.3 (blue edition; Zeiss).

### Genotyping

Post-sacrifice genotyping was performed using a piece of tissue that was placed in 75 μl extraction buffer containing 25 mM sodium hydroxide and 0.2 mM EDTA in distilled water and incubated at 98 °C for 60 min. Next, 75 μl of 40 mM tris-hydroxymethyl aminomethane-hydrochloric acid (pH 5.0) was added and centrifuged at 6000 revolutions per minute (rpm) for 3 min. The supernatant was collected for genotyping. Regarding the polymerase chain reaction (PCR), 2 μl of cDNA was mixed with 0.25 μl of MyTaq™ HS DNA Polymerase, 10 μl of 5x MyTaq™ Reaction Buffer (Bioline meridian, London, UK), 3 μl of each of the corresponding primers (0.5 μM), and 34.75 μl of distilled water (total 50 μl). Table S1 shows each primer sequence. The Cre allele was amplified using 30 cycles of 95 °C for 30 s, 61.5 °C for 30 s, and 72 °C for 60 s. For the DTA allele, there were 35 cycles of 94 °C for 30 s, 60 °C for 60 s, and 72 °C for 60 s. The PCR products were separated on 1% agarose gel and stained with GelRed® Nucleic Acid Gel Stain (Biotium, Inc. Fremont, CA, USA).

### Quantitative polymerase chain reaction

Total RNA was isolated from the ipsilateral hemisphere after HI using the RNeasy Kit (QIAGEN) and measured using a NanoDrop (Thermo Scientific). All the samples had a nucleotide ratio (A260: A280) within the range of 1.9–2.1. A total of 1 μg of extracted RNA underwent reverse transcription into cDNA using the iScript cDNA synthesis kit (Bio-Rad, Hercules, CA, USA) according to the manufacturer’s protocol. Quantitative PCR (qPCR) was performed using the Step-One-Plus Real-Time PCR machine (Applied Biosystems) with Power SYBR Green PCR Master Mix (Applied Biosystems) and the primers presented in Table S2. The set-up condition was initial denaturation at 95 °C for 5 min, subsequent denaturation at 95 °C for 5 s, annealing at 60 °C for 10 s, and elongation for 30 s for a total of 40 cycles. The cycle time values were normalized to the β-actin of the same sample. The mRNA expression levels were calculated using the delta-delta CT method as fold changes compared to the DTA^*−*^ sham samples at P13. For pre-HI insult assessments, DTA^+^ samples were compared with DTA^*−*^ samples at P10 after tamoxifen administration at P8 and P9. All samples were examined in triplicate. The aforementioned analyses were performed using the StepOne software program (version 2.3, Applied Biosystems).

### Quantitative enzyme-linked immunosorbent assay

Ipsilateral hemispheres were collected from the pups at P13 after administering the HI insult at P10. Tissue samples were homogenized in N-PER™ Neuronal Protein Extraction Reagent (Thermo Fisher) with cOmplete™, Mini, EDTA-free Protease Inhibitor Cocktail (Sigma-Aldrich) using pellet pestles, blue polypropylene (Sigma-Aldrich) on ice. After 1-h agitation at 4 °C, lysates were centrifuged at 14000 rpm for 10 min. The supernatant’s protein concentration was measured using the Pierce BCA Protein Assay kit (Thermo Scientific) according to the manufacturer’s protocol. Lysate aliquots were stored at − 80 °C for subsequent analysis. After the preparation of all the samples, the IL-10 and TGF-β levels in the lysates were measured using Mouse IL-10 Quantikine ELISA Kit (R&D system) and Mouse/Rat/Porcine/Canine TGF-beta 1 Quantikine ELISA Kit (R&D system). For TGF-β assessments, the samples were treated with 1N HCL to activate the latent TGF-β before measurement using an ELISA kit. The optical density was determined at 450 nm using a microplate reader (FLUOstar Omega, BMG LABTECH, Germany) within 15 min after stopping the reactions. All measurements were performed in duplicate.

### Statistical analysis

Values are presented as mean ± standard deviation. Statistical analysis was performed using GraphPad Prism ver. 7 (GraphPad Software, Inc., San Diego, CA, USA). One-way ANOVA was used for multiple comparisons followed by the Sidak test for determining statistical significance. Moreover, we performed two-way ANOVA with the Bonferroni multiple comparison test as the post hoc test. In non-normally distributed data, Kruskal-Wallis test was used followed by the Dunn’s comparison to be consistent with the results as non-parametric test. Statistical significance was defined at *P* < 0.05.

## Results

### Microglial depletion and repopulation using tamoxifen in *Cx3cr1*^*CreER*^*Rosa26*^*DTA*^ neonatal mice

After tamoxifen administration at P8 and P9, Iba-1^+^ cells were counted at P10, P13, P17, and P24 to evaluate microglia depletion and repopulation (Fig. 1b). At P10, over 99% of the microglia was eliminated in both sexes (striatum and hippocampus level) without significant sex differences. At P13, male DTA^+^ mice had 1% microglia at the striatum level and 2% at the hippocampus level in comparison with DTA^*−*^ mice. Similarly, female DTA^+^ mice had 1% microglia at both levels compared to DTA^*−*^ mice. However, there was rapid microglia repopulation by P17. Both male and female DTA^+^ mice showed microglial repopulation of up to approximately 60% at the striatum level and 70% at the hippocampus level; however, there were between-group differences in these ratios. At P24, there was complete microglia repopulation in mice of both sexes at both the striatum and hippocampus levels (Fig. 1c-e).

### Microglial depletion persisted after the HI insult

**Fig. 2:**
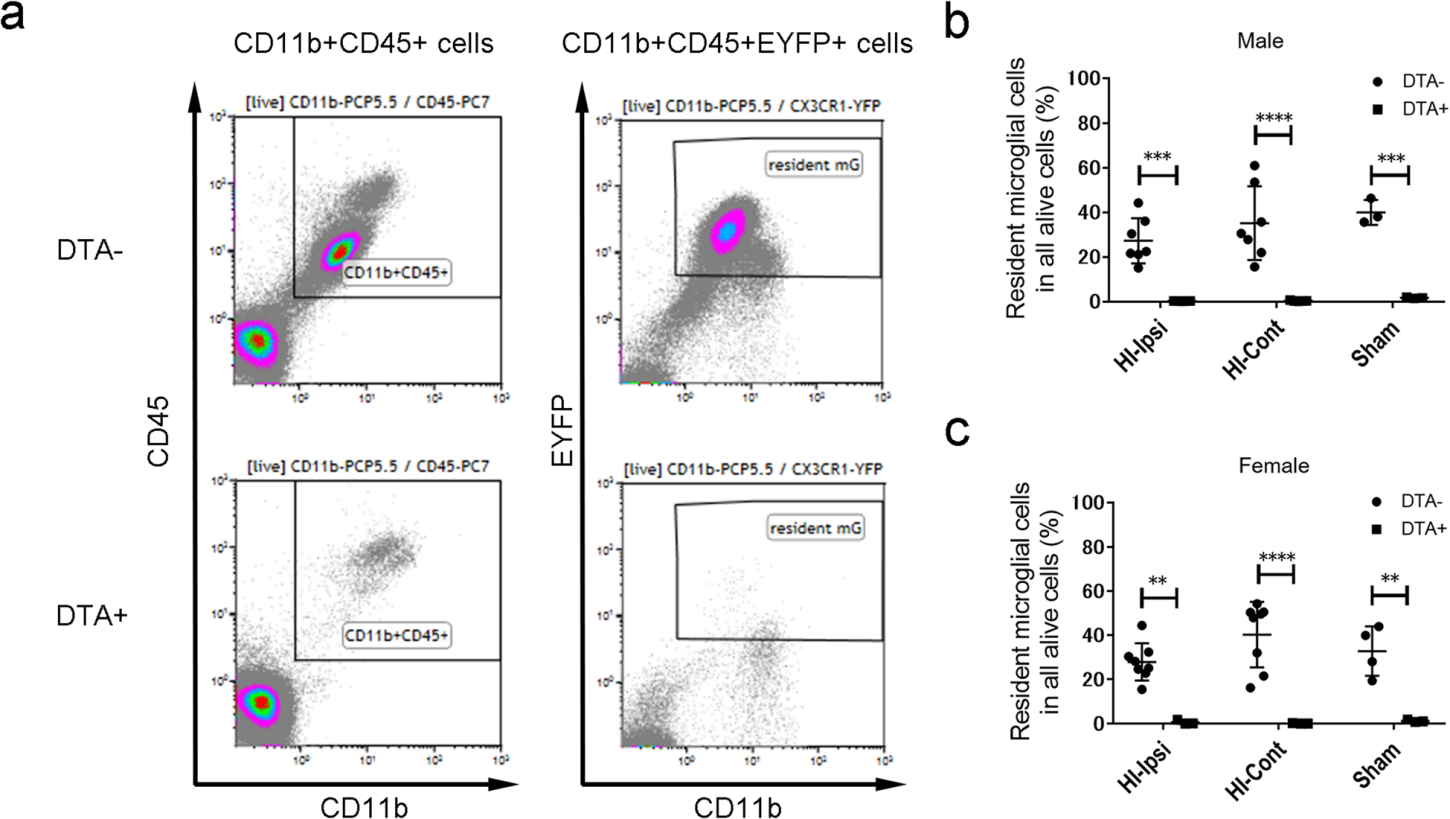
At P14, there was persisted microglial depletion following HI at P10. **a** Gating strategy for FACS analysis. After dead cell elimination, first gating was defined as CD11b^+^CD45^+^ while resident microglial cells were defined by CD11b^+^CD45^+^EYFP^+^ status. **b** Quantification of resident microglial cells in males for each group (DTA^*−*^ HI group, *n* = 7; DTA^+^ HI group, *n* = 5; DTA^*−*^ sham group, *n* = 3; DTA^+^ sham group, *n* = 3). **c** Quantification of resident microglial cells in females in each group. (DTA^*−*^ HI group, *n* = 8; DTA^+^ HI group, *n* = 3; DTA^*−*^ sham group, *n* = 4; DTA^+^ sham group, *n* = 3) **P < 0.01, ***P < 0.001, ****P < 0.0001; one-way ANOVA followed by the Sidak test. Bars reflect mean ± SD

At P13, we performed fluorescence-activated cell sorting (FACS) analyses using surface markers to determine the post-HI depletion status. We defined resident microglia as CD11b^+^CD45^+^EYFP^+^ cells and examined their ratio over the total live cell number (Fig. 2a). At P13, there were no sex differences in the differences between DTA^*−*^ and DTA^+^ mice in both the ipsilateral and contralateral sides of the brain after HI and in the sham group (Fig. 2b, c). Moreover, we performed immunohistochemistry to confirm microglia depletion and EYFP colocalization. We observed very few Iba-1^+^ cells in DTA^+^ mice with most being colocalized with EYFP (Figure S2). These findings suggested microglia depletion 3 days after HI and 4 days after the second and last tamoxifen injection.

### Microglial depletion aggravated the post-HI infarction size in male mice

**Fig. 3:**
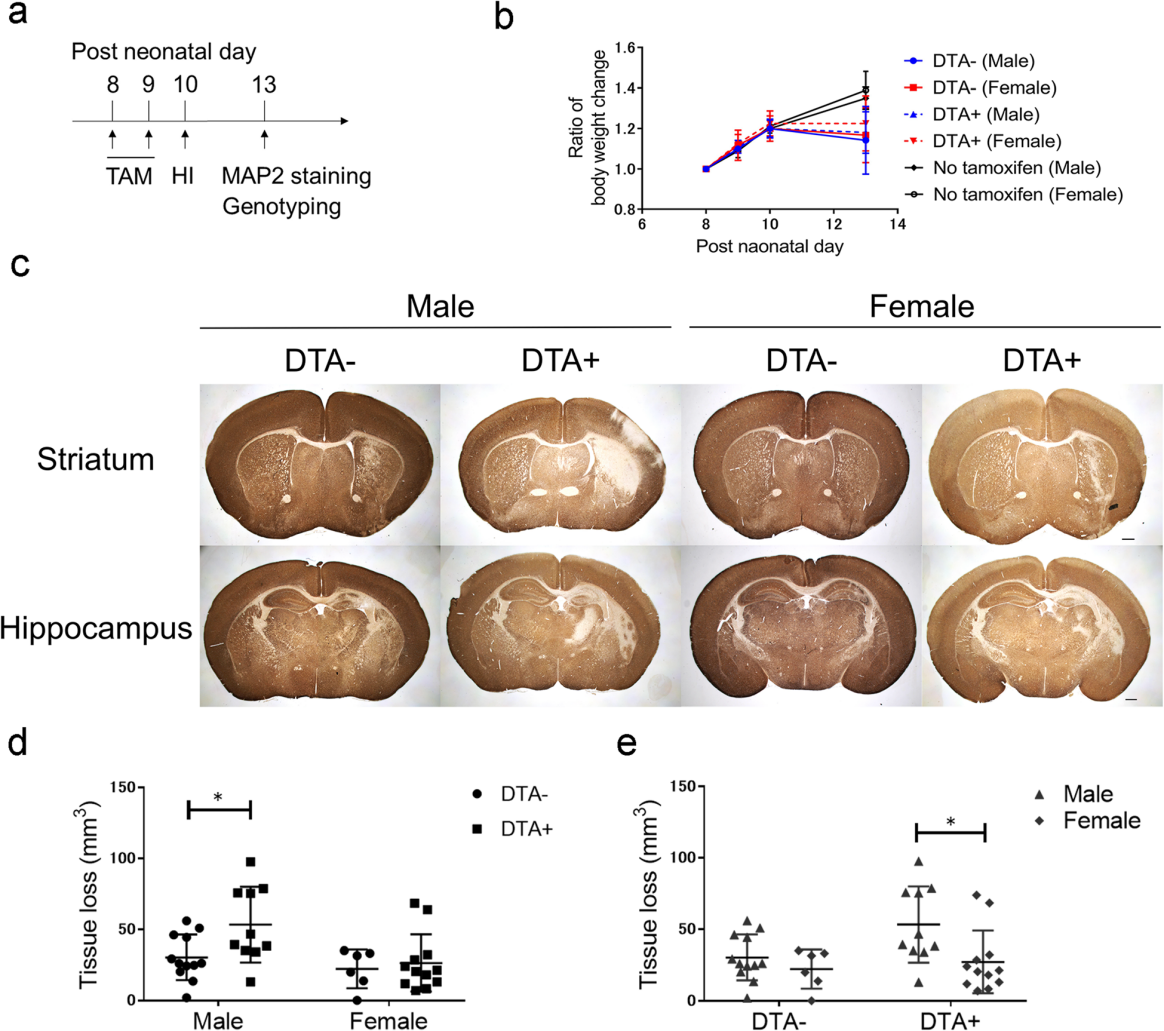
Evaluation of infarct size through MAP-2 staining at P13 following HI insult at P10. **a** The experimental design. **b** The ratio of body weight change in this experiment. **c** Representative slides of MAP-2 staining in male and female DTA^*−*^ and DTA^+^ mice. The upper and lower row shows the striatum and hippocampus level, respectively. The scale bar indicates 500 μm. **d**, **e** Quantification of infarct volume in DTA^*−*^ and DTA^+^ mice of each gender, as well as males and females of each genotype (DTA^*−*^ male, *n* = 12; DTA^+^ male, *n* = 10; DTA^*−*^ female, *n* = 6; DTA^+^ female, *n* = 12; no tamoxifen male, *n* = 6; no tamoxifen female *n* = 9) *P < 0.05; two-way ANOVA followed by the Bonferroni multiple comparison test

To assess the effect of microglia in neonatal brain injury, we performed HI at P10 after microglial depletion. The mice were sacrificed at P13 and genotyped (Fig. 3a). All the DTA groups showed stagnated weight gain during tamoxifen administration compared to the non-treated groups (Fig. 3b); moreover, there was no mortality at P13. Neuronal damage was evaluated by MAP-2 staining (Fig. 3c). There was a significant difference in tissue loss between male DTA^*−*^ and DTA^+^ mice (Fig. 3d) and between male and female DTA^+^ mice (Fig. 3e). Further, GFAP staining showed higher gliosis in DTA^+^ mice than in DTA ^-^ mice (Figure S3).

### Post-HI microglial depletion aggravated neuronal apoptosis

**Fig. 4:**
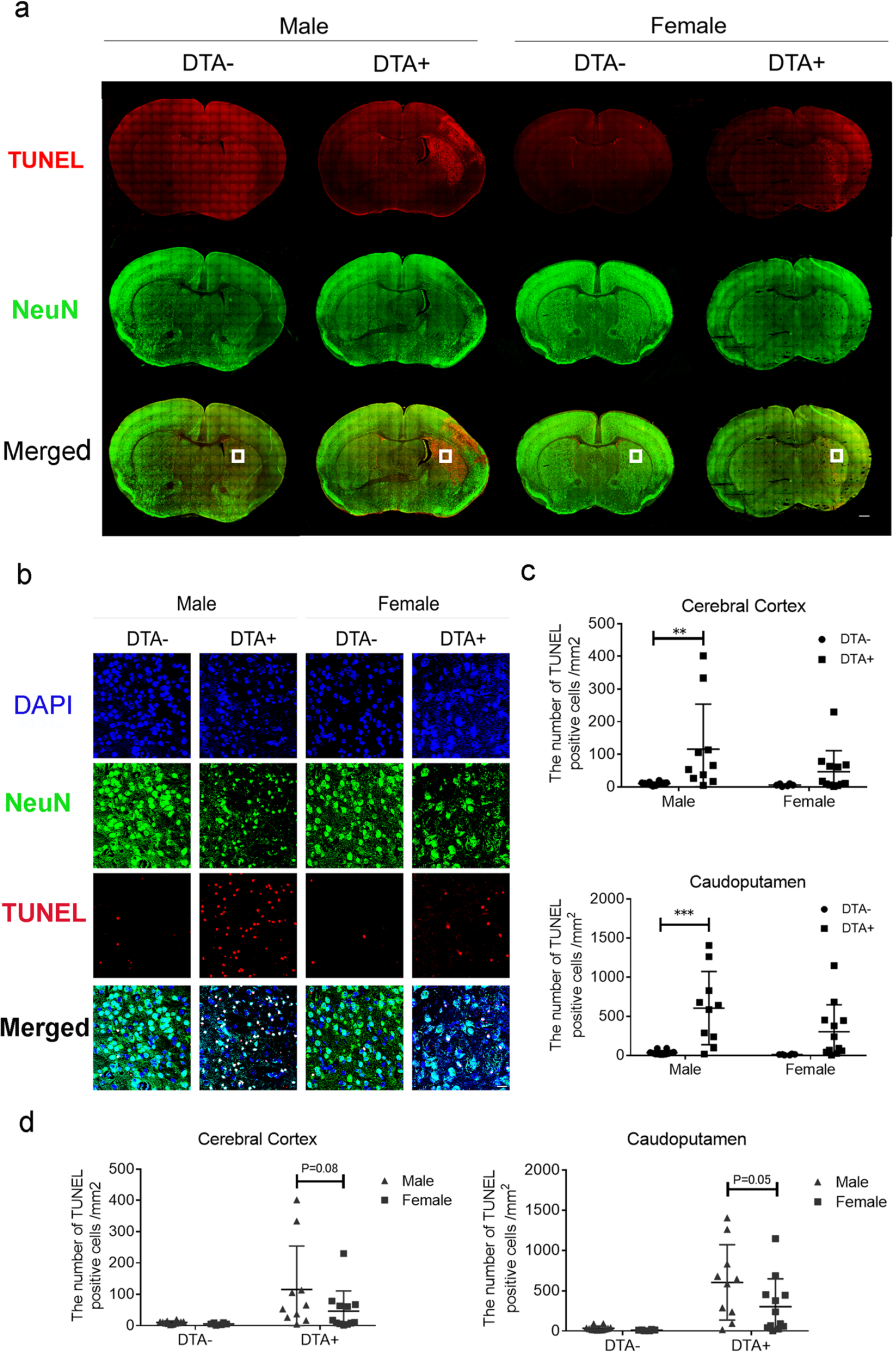
TUNEL and NeuN staining at P13 following HI insult at P10. **a** Representative image of co-immunofluorescent staining at the striatum level. The scale bar indicates 500 μm. **b** High magnification of the TUNEL^+^ area at the striatum level in each gender and genotype in the square shown in Fig. 1. Scale bar indicates 20 μm. **c** Quantification of TUNEL^+^ cells/mm^2^ in the caudoputamen and cerebral cortex. **d** Comparison of sex differences within the same genotype. The number of TUNEL-positive cells in each interest region at P13 following HI insult at P10 (DTA^*−*^ male, *n* = 12; DTA^+^ male, *n* = 10; DTA^*−*^ female, *n* = 6; DTA^+^ female, *n* = 12) **P < 0.01, ***P < 0.001. For the caudoputamen, two-way ANOVA was performed followed by the Bonferroni multiple comparison test. For the cerebral cortex, the Kruskal-Wallis test was performed followed by Dunn’s multiple comparison test. Bars depict mean ± SD

TUNEL and NeuN staining was performed in the same sections as those used for MAP-2 evaluation at P13 to assess the microglia effects on neuronal TUNEL-related apoptosis. At both the striatum and hippocampus levels, the TUNEL^+^ cluster area matched the areas without NeuN (Fig. 4a, Figure S4a) and MAP-2 (Fig. 3c) staining. High magnification demonstrated the co-localization of TUNEL^+^ and NeuN^+^ cells (Fig. 4b). Among the male mice, DTA^+^ mice showed a significantly greater TUNEL^+^ cell density than that in DTA^*−*^ mice in the caudoputamen, cerebral cortex, and thalamus (Fig. 4c, Figure S4b). Among females, DTA^+^ mice showed a significantly greater TUNEL^+^ cell density than that in DTA^*−*^ mice in the hippocampus and thalamus (Figure S4b). Assessment of sex differences in parallel genotypes showed a tendency of exacerbated apoptosis among males compared to that in females except for in the hippocampus (Fig. 4d, S4c).

### Post-HI inflammation-related gene expression at P13

**Fig. 5:**
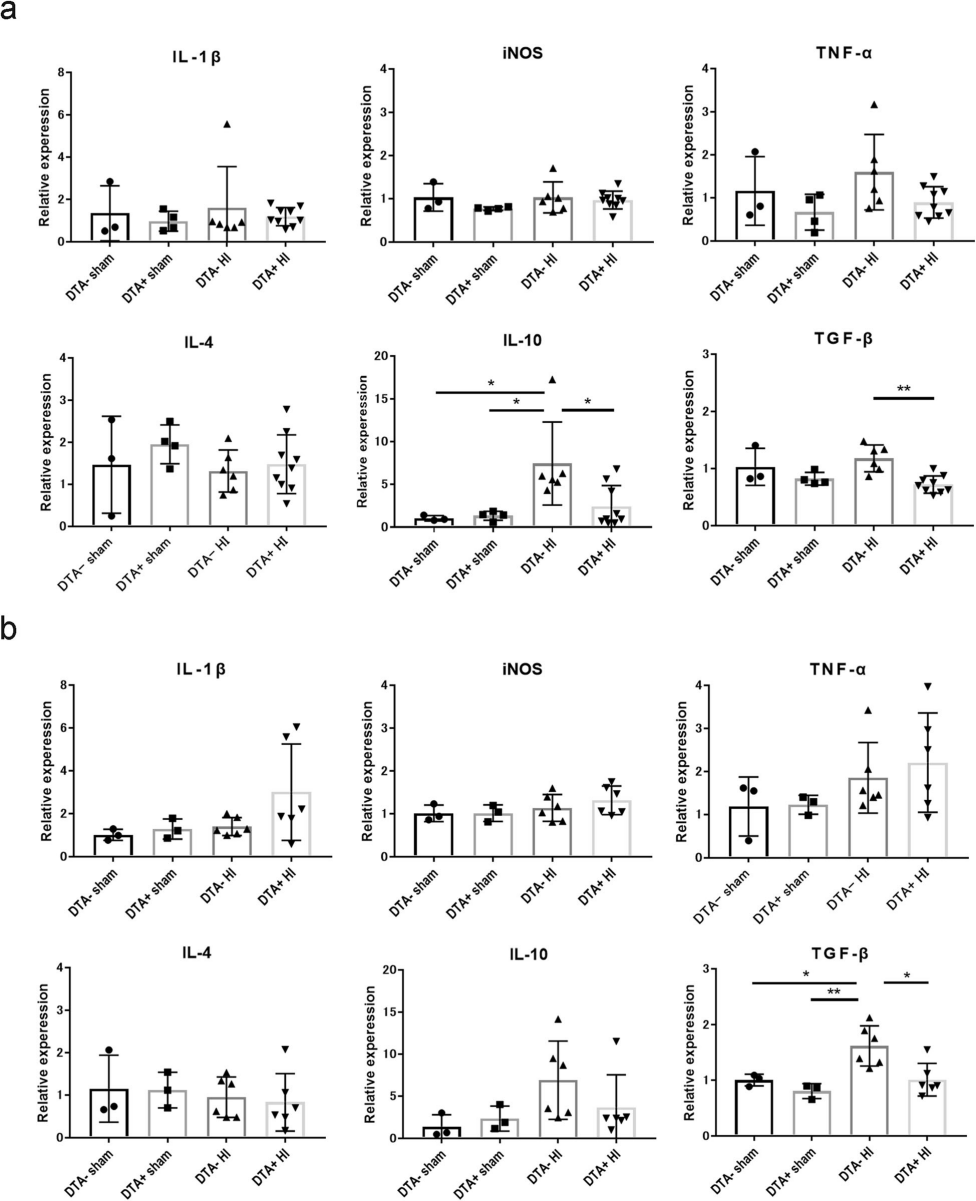
Pro- and anti-inflammatory gene expression at P13 following HI insult at P10. Relative expression of pro-inflammatory (IL-1β, TNF-α, and iNOS) and anti-inflammatory genes (IL-4, IL-10, and TGF-β) was measured by quantitative PCR at P13 in males (**a**) (DTA^*−*^ sham, *n* = 3; DTA^+^ sham, *n* = 4; DTA^*−*^ HI, *n* = 6; DTA^+^ HI, *n* = 9) and females (**b**) (DTA^*−*^ sham, *n* = 3; DTA^+^ sham, *n* = 3; DTA^*−*^ HI, *n* = 6; DTA^+^ HI, *n* = 6). All values were normalized to β-actin. The expression levels of the target mRNAs were calculated and compared with the DTA^*−*^ and sham groups. Values are presented as mean ± SD. *P < 0.05, **P < 0.01. Regarding TNF-α, iNOS, IL-4, and TGF-β expression in males and IL-1β, iNOS, IL-4, and TGF-β expression in females; one-way ANOVA followed by the Sidak multiple comparison test. Regarding IL-β and IL-10 in males and TNF-α and IL-10 in females; Dunn’s multiple comparison test

To investigate whether microglial depletion has an effect on pro- and anti-inflammatory cytokine expression at P13 following HI at P10, we performed quantitative PCR using homogenates of the ipsilateral hemisphere. Among males, there was significantly suppressed IL-10 and TGF-β gene expression in the DTA^+^ HI group compared to in the DTA^*−*^ HI group (Fig. 5a). There were no differences in IL-1β, TNF-α, iNOS, and IL-4 expressions (Fig. 5a). Among females, only TGF-β expression showed a significant difference between the DTA^*−*^ HI and DTA^+^ HI groups (Fig. 5b). Contrastingly, there were no differences in inflammatory and anti-inflammatory cytokine expression at P10 following tamoxifen administration at P8 and P9 (Figure S5a).

### Microglial depletion attenuated IL-10 and TGF-β protein levels

**Fig. 6:**
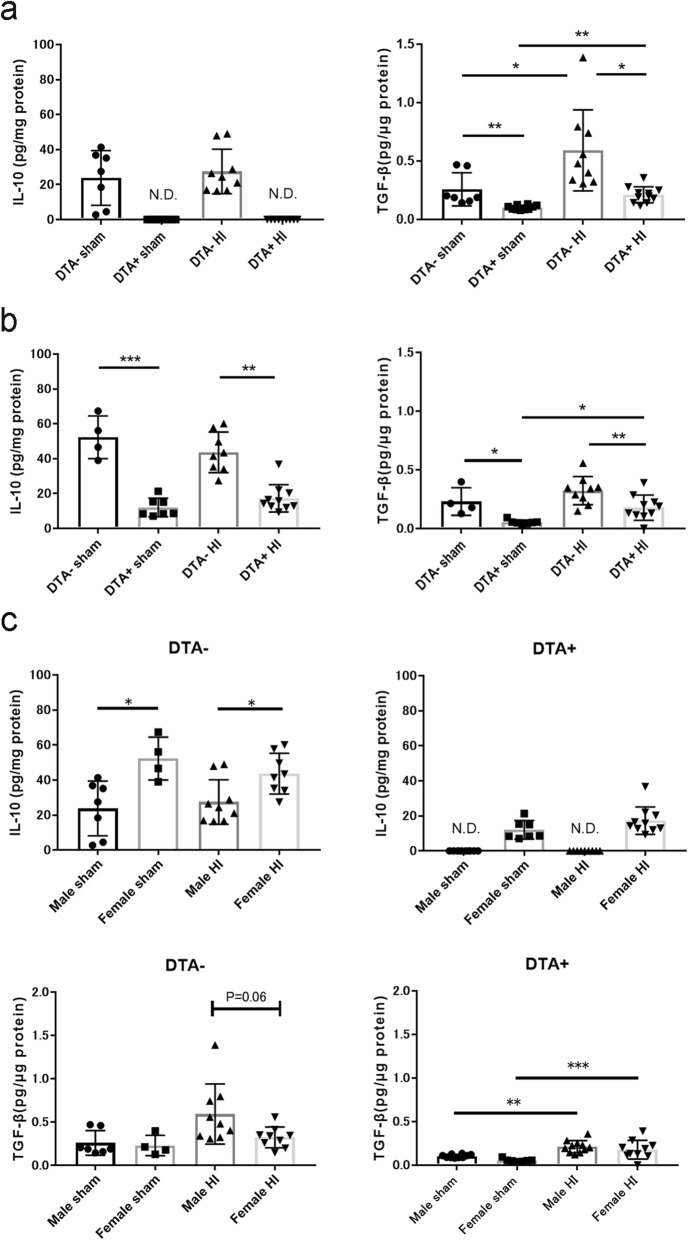
ELISA quantification of IL-10 and TGF-β protein levels at P13 following HI insult at P10. **a** Males (DTA^–^ sham, *n* = 7; DTA^+^ sham, *n* = 10; DTA^*−*^ HI, *n* = 9; DTA^+^ HI, *n* = 9). **b** Females (DTA^*−*^ sham, *n* = 4; DTA^+^ sham, *n* = 7; DTA^*−*^ HI, *n* = 9; DTA^+^ HI, *n* = 10). **c** Comparison of sex differences within the same genotype. *P < 0.05, **P < 0.01, ***P < 0.001. Kruskal-Wallis test followed by Dunn’s multiple comparison test. Bars depict mean ± SD

Based on the quantitative PCR results, the IL-10 and TGF-β protein levels were measured using ELISA in the ipsilateral hemispheres at P13 following HI at P10. Compared to male mice in the DTA^+^ group, those in the DTA^*−*^ group showed higher IL-10 expression levels (Fig. 6a). There were significant differences in the TGF-β levels between the DTA^*−*^ and DTA^+^ groups comprised of both sham and HI mice (Fig. 6a). Moreover, female mice in the DTA^*−*^ and DTA^+^ groups comprised of both sham and HI mice had significantly different IL-10 and TGF-β expression levels (Fig. 6b). We observed sex differences in IL-10 expression regardless of genotype. Moreover, male mice in the DTA^*−*^ group showed higher TGF-β expression compared to female mice in this group. However, there were no corresponding sex differences between male and female mice in the DTA^+^ group (Fig. 6c).

## Discussion

In this study, we report a microglial depletion model in early postnatal life that aggravated neuronal damage after HI injury. Microglial depletion reduced IL-10 and TGF-β levels both under normal and post-HI (more pronounced) conditions. There was a sex-specific sensitivity where males were more affected than females.

To our knowledge, this is the first report on the effect of microglia in neonatal HIE using a neonatal genetic microglial-depletion model. We recently demonstrated competitive CNS repopulation following microglia depletion and the development of fatal demyelinating disease using an acute microglial-depletion *Cx3cr1*^*CreER*^*Rosa26*^*DTA*^ model [23, 41]. This genetic microglial-depletion model is suitable for neonatal experiments. Furthermore, we did not observe found BBB disruption after microglial depletion [41]. However, this genetic model requires tamoxifen administration for microglia depletion. Tamoxifen has a neuroprotective effect involving inhibition of neuronal nitric oxidase synthase, scavenging of reactive oxygen species, and the estrogen effect [49, 50]. Despite these model limitations, we believe that they caused an insignificant bias between the DTA^*−*^ and DTA^+^ groups given that both groups received similar tamoxifen doses.

Previous studies have employed microglial-depletion models of adult stroke, Alzheimer’s disease, and retinal disease [26, 28, 30, 33, 51, 52]. To study the early-life role of microglia, an acute microglial depletion model was required. Intracerebral administration of clodronate encapsulated into liposomes (Clod-lip) has been shown to immediately induce microglia depletion [53]. However, this method causes unselective cell damage, including blood vessel damage and incomplete depletion [22, 33]. Among adult mice, *Cx3cr1*^*CreER*^*Rosa26*^*DTR*^ mice have been reported as an acute microglial depletion model through tamoxifen administration with subsequent diphtheria toxin administration [31, 54]. To avoid administering pups with multiple injections, we employed the *Cx3cr1*^*CreER*^*Rosa26*^*DTA*^ strain.

First, we confirmed microglia depletion and repopulation in our neonatal model. At P10, compared with DTA^*−*^ mice, the efficiency of resident microglial depletion was > 99% in DTA^+^ mice. This depletion status remained until at least P13. At P17, we observed microglia repopulation clusters (Figure S6), which is consistent with previous findings on adult microglial depletion models [54]. These clusters were few and asymmetrically distributed throughout the brain. This asymmetrical distribution could induce significant result variations if the analysis is conducted during repopulation; therefore, we evaluated brain damage at P13. Compared to the DTA-p24 mice, there was a numerical, but not significant, higher repopulation in the DTA+p24 mice. This is consistent with a previous report that the number of repopulated microglia at day 7 was higher after the withdrawal of a selective CSF1R inhibitor than in the normal brain [24]. We assumed that the proliferation ability of repopulated microglia was higher than that of normal microglia. Next, we confirmed that these depletion conditions remained after HI. We identified resident microglia with EYFP using FACS and recorded a high depletion rate (97%) after 4 days (P13), which covered the time period of the HI insult and recovery. Subsequently, we confirmed microglia depletion and EYFP colocalization through immunohistochemistry (Figure S2). To assess the exclusive effect of microglial depletion, we conducted the quantitative PCR of the brain after tamoxifen administration. Figure S5a shows that there was no difference between DTA^*−*^ and DTA^+^ mice; however, DTA^+^ mice showed a tendency of lower IL-1β expression compared to DTA^*−*^ mice. Further, we stained astrocytes in both mice groups using GFAP for slides after tamoxifen administration at P8 and P9. Moreover, Figure S5b showed no between-group differences in the astrocytes.

Given that there was little between-genotype difference in the inflammation and astrocyte reaction before the HI insult, our findings indicate that microglial depletion could lead to aggravation of neuronal damage and astrocyte reaction (Fig. 3, Figure S3). Regarding oligodendrocytes, we observed MBP loss in the striatum but not the hippocampus level of the DTA^+^ mice (Figure S7). This is consistent with previous findings of a neonatal depletion model using Clod-lip, which demonstrated that microglia depletion increased the brain injury severity and volume through middle cerebral artery occlusion (MCAO) [53]. Moreover, an adult depletion model demonstrated that microglia depletion enhances the post-MCAO size of ischemic brain injury at 24 h [26]. Further, our findings indicated a sex difference in the effect of microglial depletion after HIE. Only males showed a significant difference in the MAP-2 evaluation of the brain injury after microglia depletion. TUNEL staining showed sex-specific effects on different regions. There have been several reports regarding sex- and region-specific effects following HI insult from the perspective of mitochondrial metabolism and apoptotic mechanism [55–57]. Further, there have been previous reports on sex differences in the microglia number and function [40, 58]. These overall differences might contribute toward TUNEL staining differences. There have been recent studies on sex differences in brain inflammation and microglial effects [38–40]. For example, microglia in males have been reported to have higher migrational capacity than those in females, which could be related to the protective effect of microglia depletion [40]. Alternatively, the sex differences in our model could be attributed to the estrogen effect of tamoxifen. Estrogen has neuroprotective effects in the ischemic brain, which are stronger in females given the more effective estrogen receptor upregulation [59–61].

Previous studies on microglial depletion in adult stroke models reported significantly increased inflammatory mediator levels in injured tissues at 24 h following MCAO [26]. In our study, analyses at 72 h after the injury showed no significant differences in the levels of several pro-inflammatory cytokines. This could be attributed to our experimental design of the evaluation of pro-inflammatory cytokines at the end of the sub-acute phase [3, 62]. We found differences in the anti-inflammatory cytokines between DTA^*−*^ and DTA^+^ mice. We found suppressed IL-10 upregulation and TGF-β downregulation in microglia-depleted mice 3 days after the HI insult. Microglia have been shown as a major post-stroke source of TGF-β [63]. To confirm these quantitative PCR results at the protein level, we conducted ELISA for IL-10 and TGF-β and found that they were significantly decreased in the DTA^+^ and sham group (Fig. 6a, b). With respect to sex differences in IL-10, there was a tendency of lower expression in males than in females regardless of the genotype (Fig. 6c). Interestingly, we did not detect IL-10 in microglia eliminated male model. These findings suggest that IL-10 production is dependent on microglia in males. With respect to TGF-β, there were sex differences in the DTA^*−*^ mice and DTA^+^ mice (Fig. 6c), which suggested that TGF-β is mainly dependent on microglia especially in males. Previous studies have also indicated that microglia is a major source of locally produced TGF-β after HI insult [63]. However, consistent with our findings, other cells have been reported to be produced after microglia depletion [64]. Taken together, our findings indicate that microglial depletion aggravates neuronal death due to a lack of anti-inflammatory cytokines. However, other sex-related mechanisms may inhibit neuronal damage in females, including the mitochondrial respiratory capacity of astrocytes, as well as the different contribution of TGF-β by resident microglia [55, 65, 66]. Consistent with our findings, Jin reported that microglial depletion increased ischemia-induced astrocytic inflammatory response and neural injury [26]. Therefore, assessing astrocytic responses is a potential avenue to understand the impact of microglia depletion in neonatal hypoxic-ischemic mice.

## Conclusions

In this study, we established a model of 99% microglial depletion in neonatal mice. Our findings indicate that resident microglia play a neuroprotective role early after neonatal hypoxic ischemia, which is predominant in males with the protective effect involving anti-inflammatory cytokines.

## Supplementary information

**Additional file 1: Figure S1.** Occlusion procedure by electronic coagulation of the left common carotid artery. **a** Separation from the vagus nerve. **b** The artery was grasped by bipolar electronic micro forceps. **c** Coagulation by bipolar electronic micro forceps. **d** Occlusion by electronic coagulation.

**Additional file 2: Figure S2.** Representative slide for microglia depletion and Iba-1^+^/Cx3Cr1-EYFP^+^ cell co-localization at P13 after HI at P10. The scale bar in high magnification is 20 μm.

**Additional file 3: Figure S3.** Representative GFAP staining slides. **a** Low magnification in DTA^*−*^ and DTA^+^ mice of both genders. The upper row shows the striatum level while the lower row shows the hippocampus level. Scale bar indicates 500 μm. The square indicates the higher magnification. **b** High magnification of GFAP staining. Scale bar indicates 100 μm.

**Additional file 4: Figure S4.** TUNEL and NeuN staining at P13 following HI insult at P10. **a** Representative image of co-immunofluorescent staining at the hippocampus level. Scale bar indicates 500 μm. **b** Quantification of TUNEL^+^ cells/mm^2^ in the hippocampus and thalamus. **c** Comparison of sex differences within the same genotype. The number of TUNEL positve cells in each interest region at P13 following HI insult at P10. (DTA^*−*^ male, *n* = 12; DTA^+^ male, *n* = 10; DTA^*−*^ female, *n* = 6; DTA^+^ female, *n* = 12) *P < 0.05, ****P < 0.001. The Kruskal-Wallis test followed by Dunn’s multiple comparison test. Bars depict mean ± SD.

**Additional file 5: Figure S5. a** Cytokine analysis at P10 after tamoxifen administration at P8 and P9. (DTA^*−*^ male, *n* = 5; DTA^+^ male, *n* = 3; DTA^*−*^ female, *n* = 4; DTA^+^ female, *n* = 4) Bars depict mean ± SD. **b** GFAP staining at P10 after tamoxifen administration at P8 and P9. CA; cornu ammonis DG; dentate gyrus.

**Additional file 6: Figure S6.** Uneven repopulation at P17 following tamoxifen administration at P8 and P9. **a** There was an equal number of Iba-1 staining cells in the whole in DTA^*−*^ mice. **b** In the DTA^+^ mice, there was partial detection of Iba-1 staining cells with uneven distribution. Scale bar indicates 500 μm.

**Additional file 7: Figure S7.** Representative slides of MBP staining. **a** Striatum level. **b** Hippocampus and thalamus level. Cont; contralateral side (healthy side) Ipsi; Ipsilateral side (injury side). Scale bar indicates 500 μm.

**Additional file 8: Table S1.** Genotyping Primer.

**Additional file 9: Table S2.** qPCR Primer.

## Data Availability

All data used in this manuscript are available from the corresponding author upon reasonable request.

## Abbreviations

HIE: Hypoxic-ischemic encephalopathy
IL-1β: Interleukin
TNF-α: Tumor necrosis factor
TGF-β: Tumor necrosis factor
BBB: Blood-brain barrier
EYFP: Enhanced yellow fluorescent protein
PFA: Paraformaldehyde
FACS: Fluorescence-activated cell sorting
EDTA: Ethylenediaminetetraacetic acid
TUNEL: Transferase-dUTP nick end labeling
PCR: Polymerase chain reaction
ELISA: Enzyme-linked immunosorbent assay
Clod-lip: Clodronate encapsulated into liposomes
HBSS: Hank’s Balanced Salt Solution
