# Supplementary figures and images for: Aggravated brain injury after neonatal hypoxic ischemia in microglia-depleted mice
Source: J Neuroinflammation. 2020 Apr 11;17:111. doi: 10.1186/s12974-020-01792-7 (PMC7149909; doi:10.1186/s12974-020-01792-7)

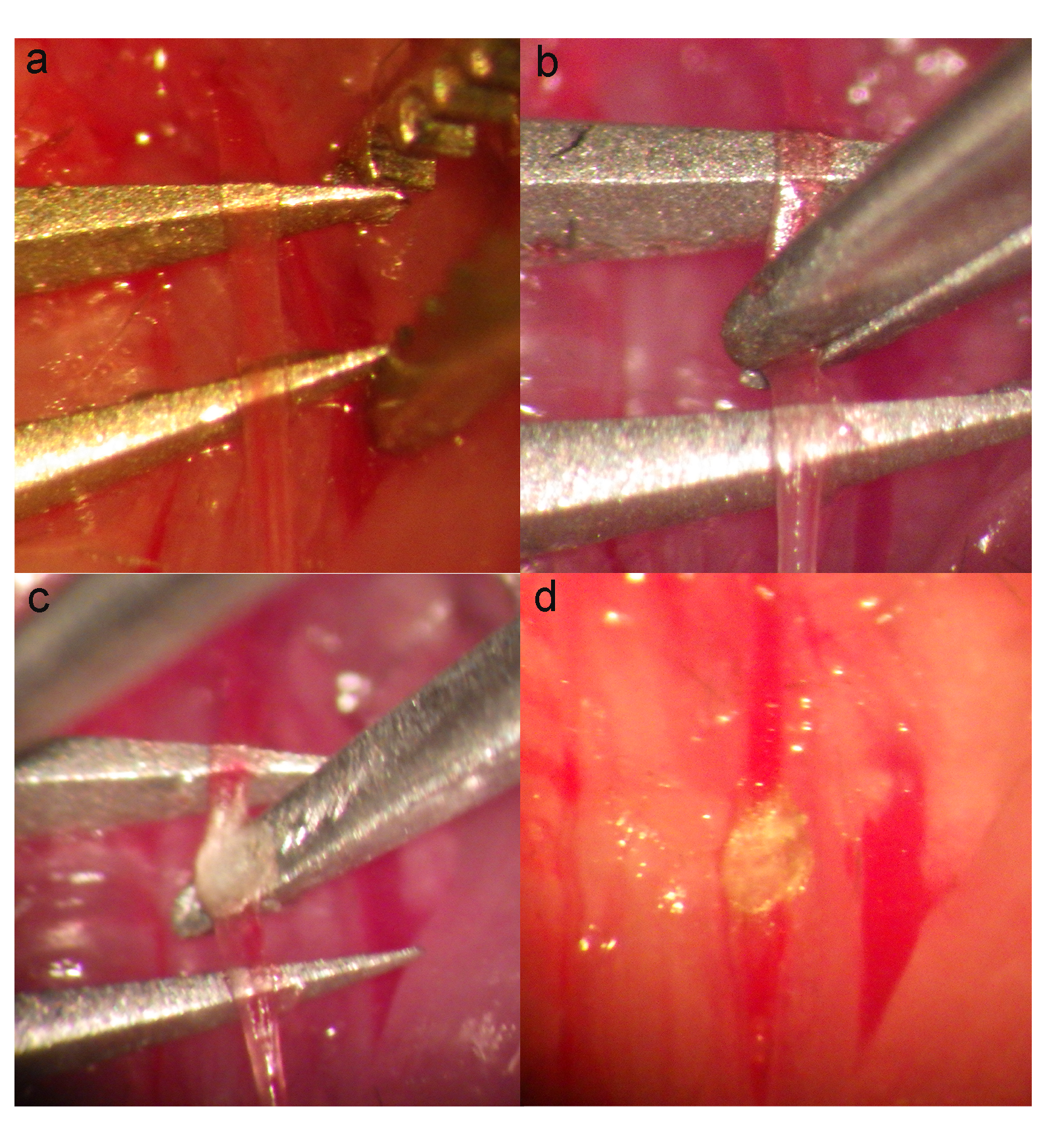

Supplement: Supplementary file 1 — Additional file 1: Figure S1. Occlusion procedure by electronic coagulation of the left common carotid artery. a Separation from the vagus nerve. b The artery was grasped by bipolar electronic micro forceps. c Coagulation by bipolar electronic micro forceps. d Occlusion by electronic coagulation. [file 12974_2020_1792_MOESM1_ESM.tif]

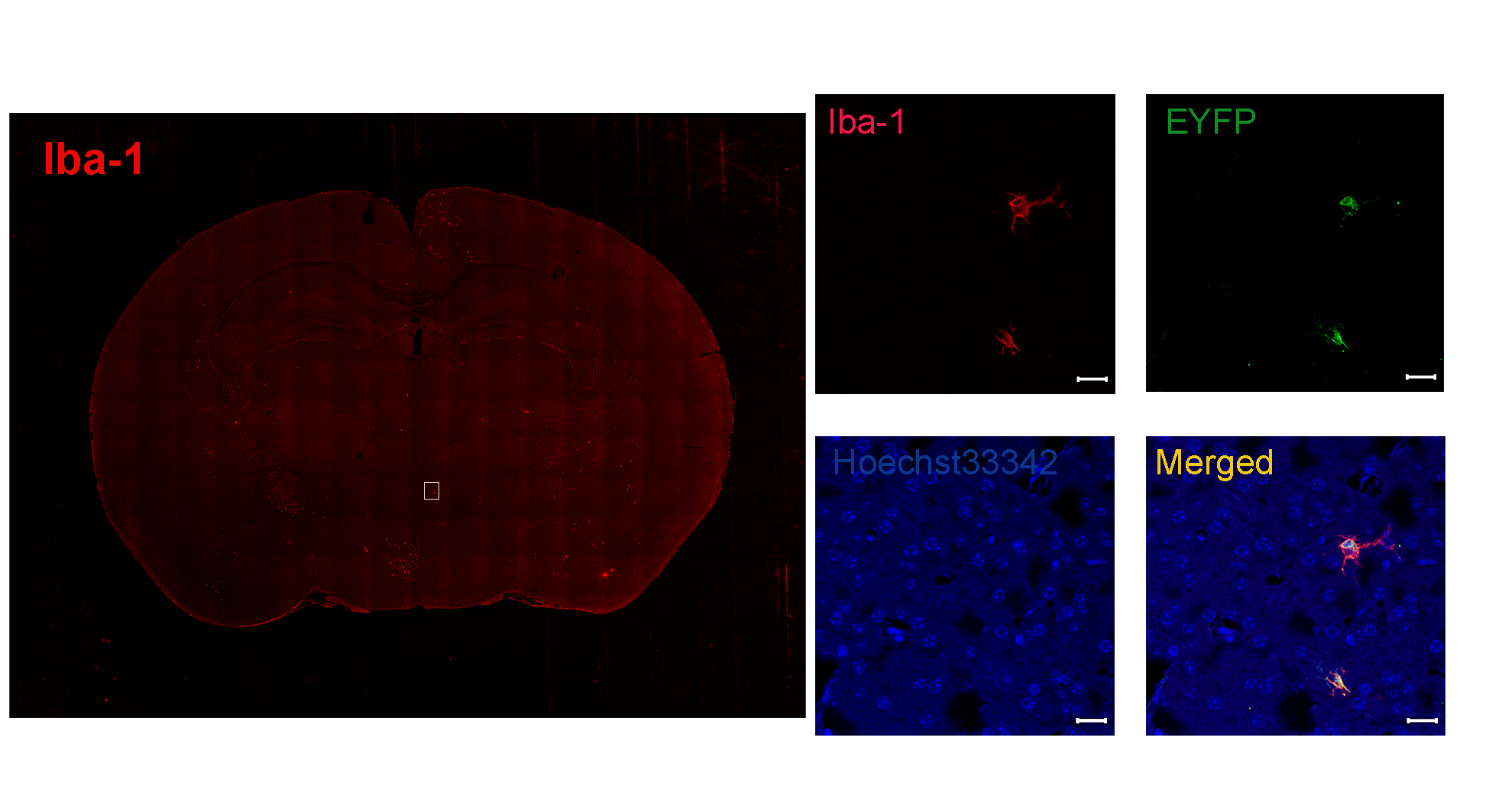

Supplement: Supplementary file 2 — Additional file 2: Figure S2. Representative slide for microglia depletion and Iba-1+/Cx3Cr1-EYFP+ cell co-localization at P13 after HI at P10. The scale bar in high magnification is 20 μm. [file 12974_2020_1792_MOESM2_ESM.tif]

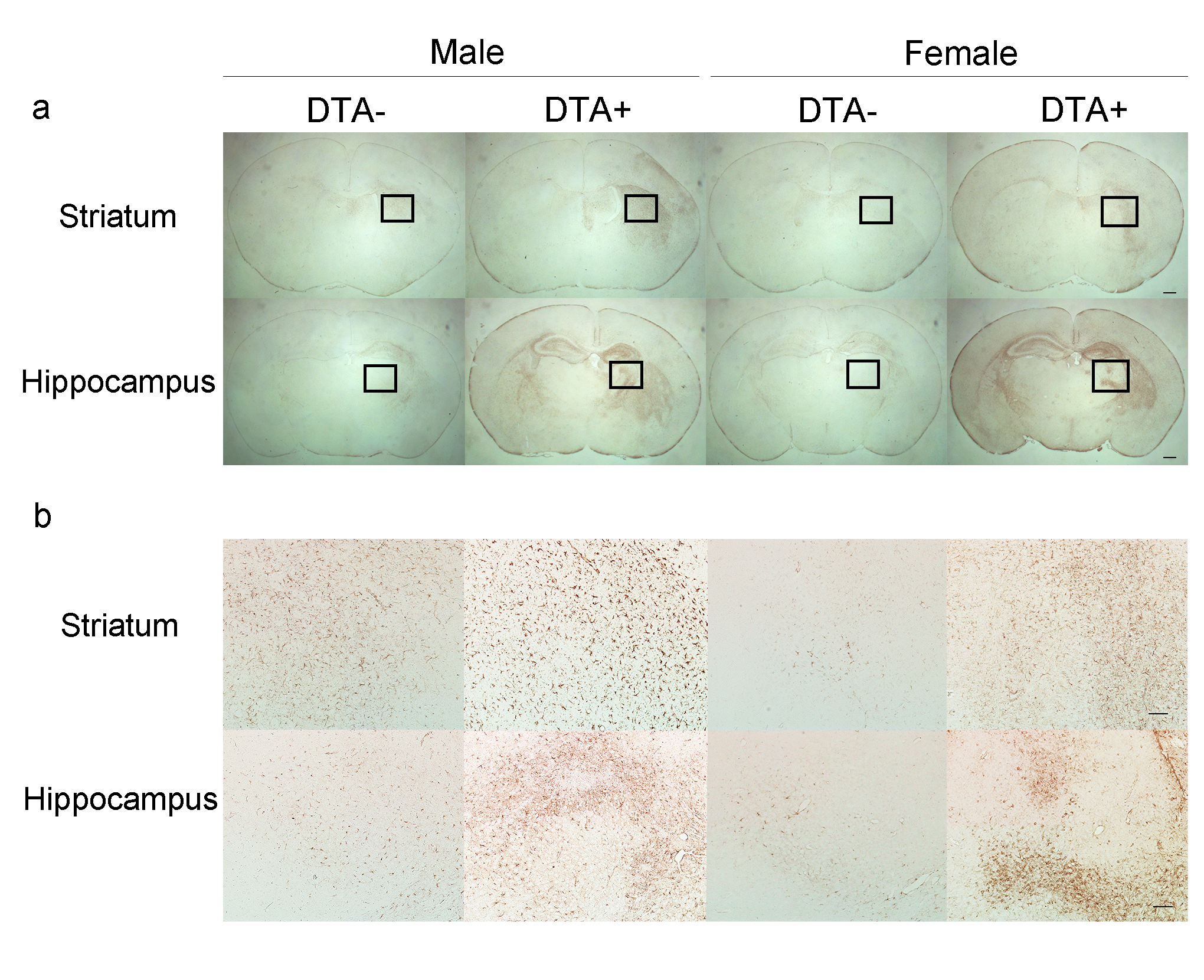

Supplement: Supplementary file 3 — Additional file 3: Figure S3. Representative GFAP staining slides. a Low magnification in DTA− and DTA+ mice of both genders. The upper row shows the striatum level while the lower row shows the hippocampus level. Scale bar indicates 500 μm. The square indicates the higher magnification. b High magnification of GFAP staining. Scale bar indicates 100 μm. [file 12974_2020_1792_MOESM3_ESM.tif]

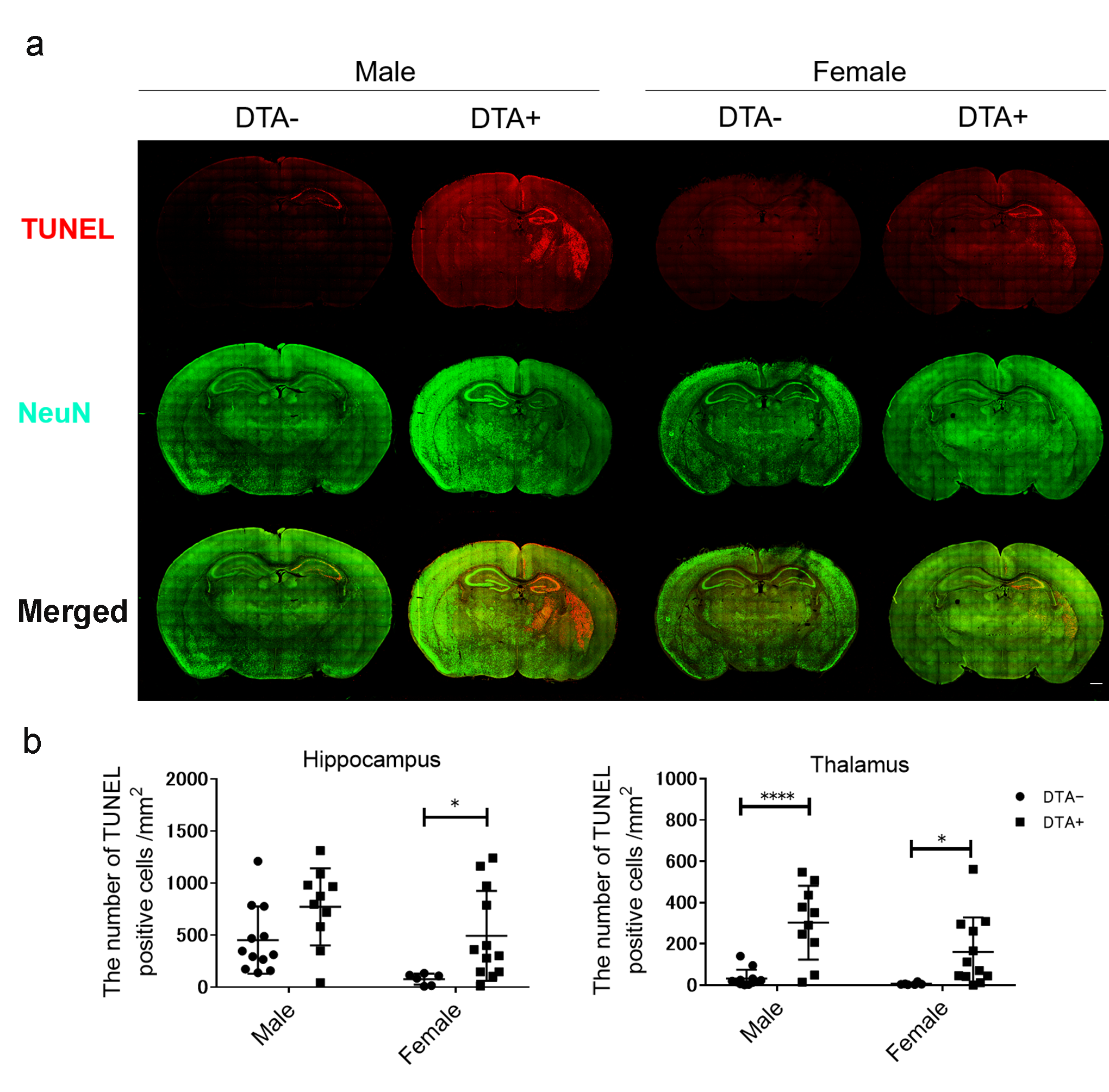

Supplement: Supplementary file 4 — Additional file 4: Figure S4. TUNEL and NeuN staining at P13 following HI insult at P10. a Representative image of co-immunofluorescent staining at the hippocampus level. Scale bar indicates 500 μm. b Quantification of TUNEL+ cells/mm2 in the hippocampus and thalamus. c Comparison of sex differences within the same genotype. The number of TUNEL positve cells in each interest region at P13 following HI insult at P10. (DTA− male, n = 12; DTA+ male, n = 10; DTA− female, n = 6; DTA+ female, n = 12) *P < 0.05, ****P < 0.001. The Kruskal-Wallis test followed by Dunn’s multiple comparison test. Bars depict mean ± SD. [file 12974_2020_1792_MOESM4_ESM.tif]

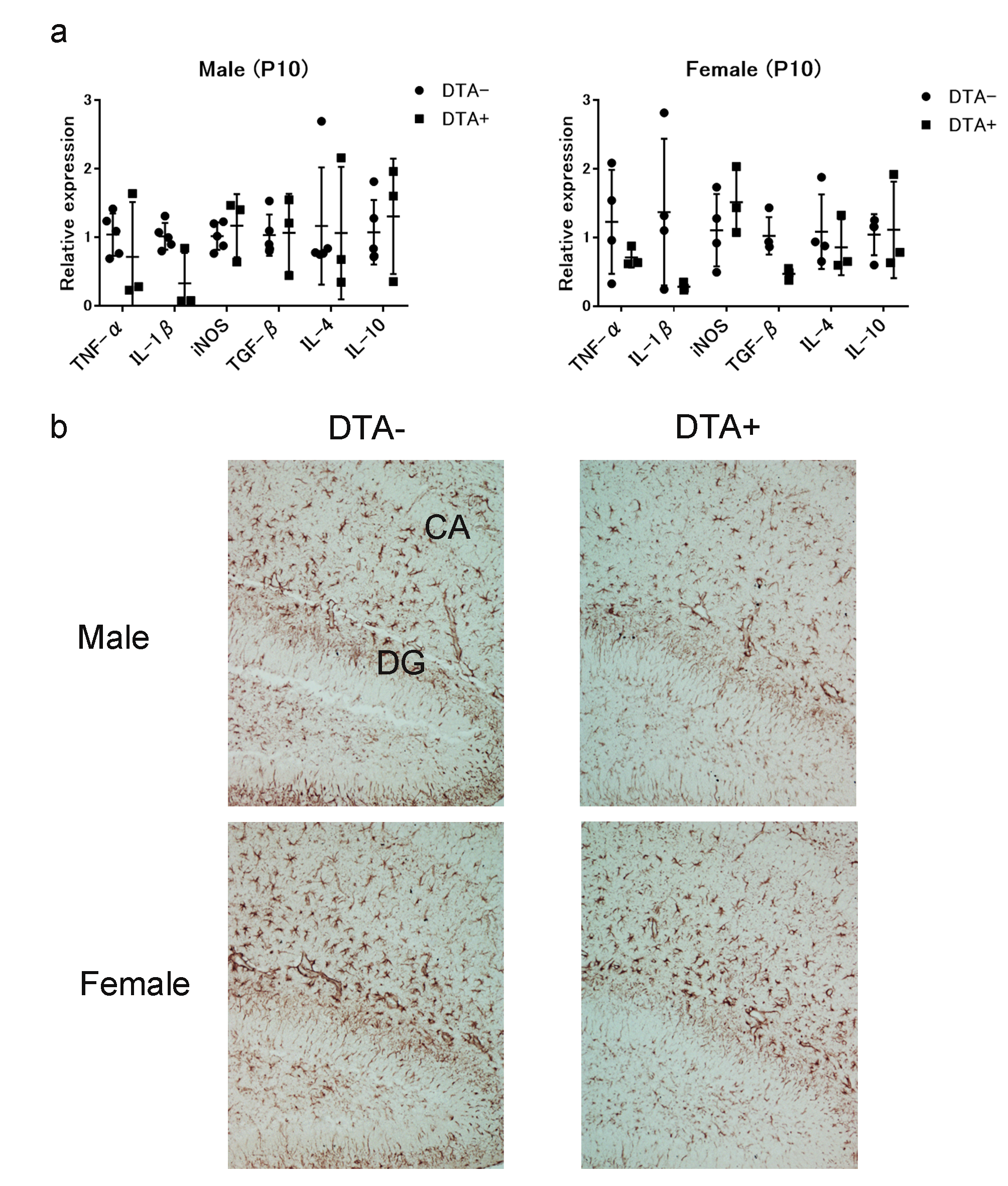

Supplement: Supplementary file 5 — Additional file 5: Figure S5. a Cytokine analysis at P10 after tamoxifen administration at P8 and P9. (DTA− male, n = 5; DTA+ male, n = 3; DTA− female, n = 4; DTA+ female, n = 4) Bars depict mean ± SD. b GFAP staining at P10 after tamoxifen administration at P8 and P9. CA; cornu ammonis DG; dentate gyrus. [file 12974_2020_1792_MOESM5_ESM.tif]

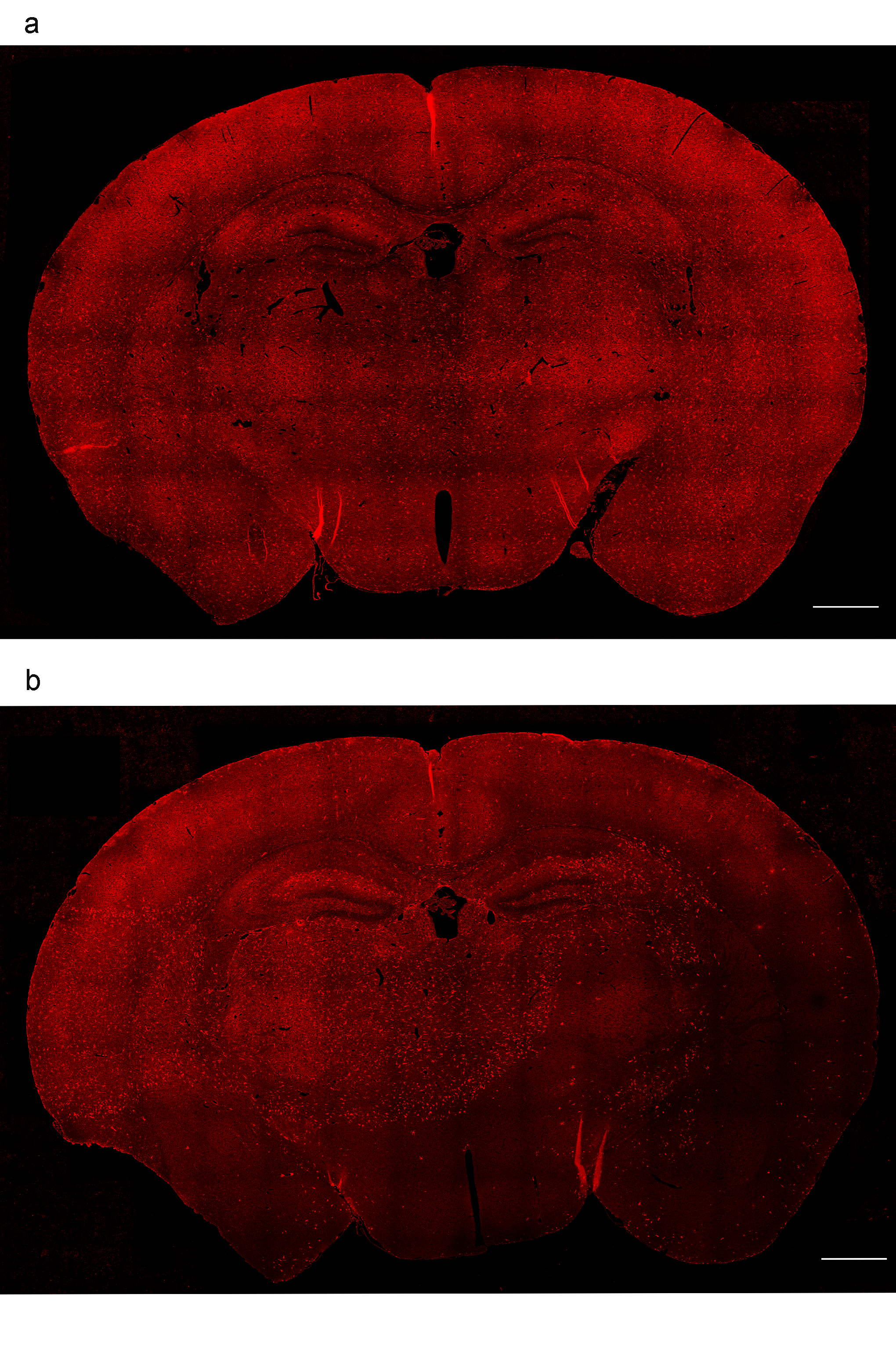

Supplement: Supplementary file 6 — Additional file 6: Figure S6. Uneven repopulation at P17 following tamoxifen administration at P8 and P9. a There was an equal number of Iba-1 staining cells in the whole in DTA− mice. b In the DTA+ mice, there was partial detection of Iba-1 staining cells with uneven distribution. Scale bar indicates 500 μm. [file 12974_2020_1792_MOESM6_ESM.tif]

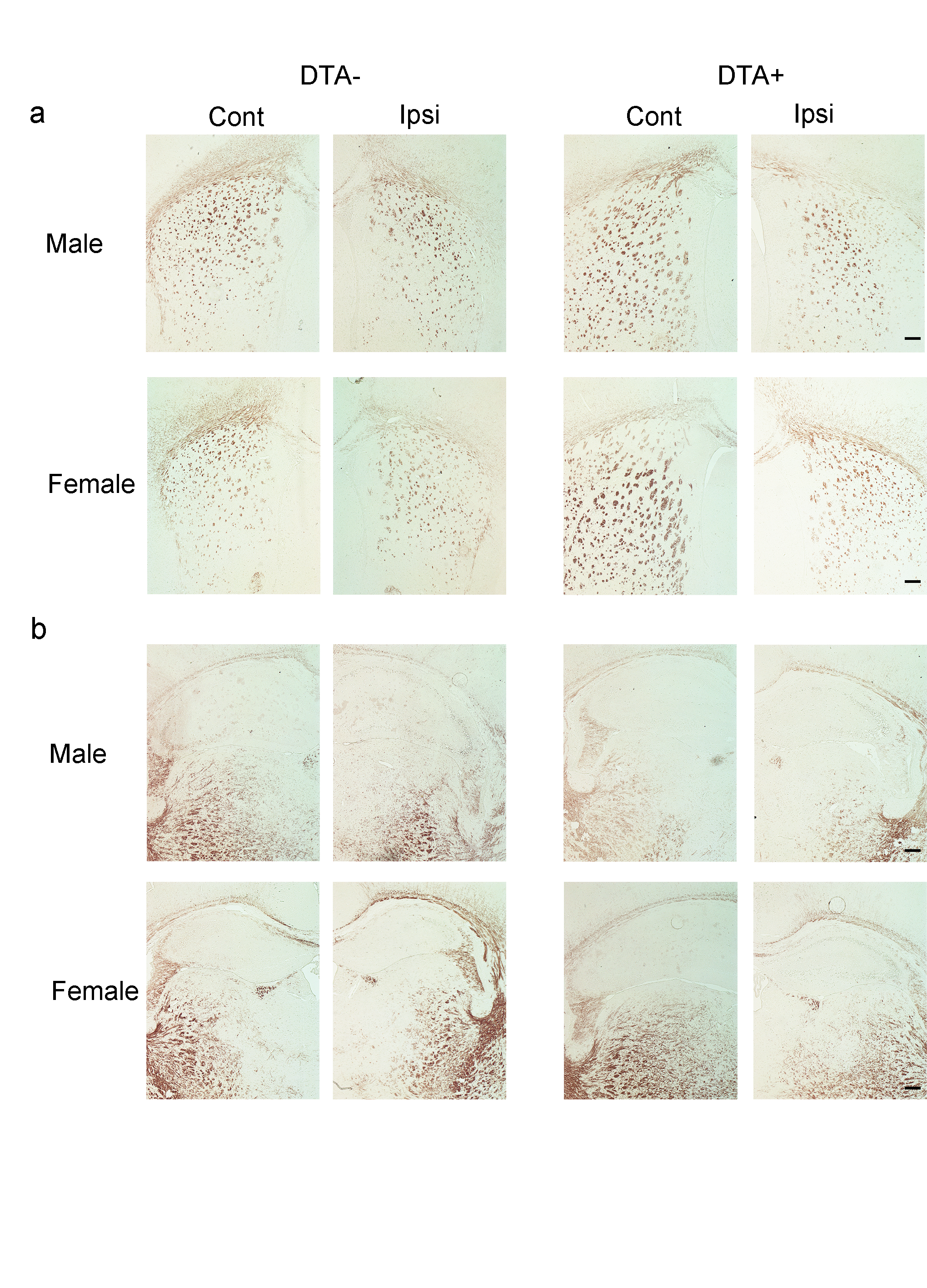

Supplement: Supplementary file 7 — Additional file 7: Figure S7. Representative slides of MBP staining. a Striatum level. b Hippocampus and thalamus level. Cont; contralateral side (healthy side) Ipsi; Ipsilateral side (injury side). Scale bar indicates 500 μm. [file 12974_2020_1792_MOESM7_ESM.tif]
